# Voriconazole injection use in patients receiving continuous renal replacement therapy

**DOI:** 10.3389/fphar.2025.1736767

**Published:** 2025-12-18

**Authors:** Ling Zhou, Zhenwei Yu, Xia Wang, Huaiyu Zheng

**Affiliations:** 1 Department of Pharmacy, Zhoushan Hospital, Zhoushan, China; 2 Department of Pharmacy, Sir Run Run Shaw Hospital, School of Medicine, Zhejiang University, Hangzhou, China; 3 Department of Pharmacy, The Affiliated Taian City Central Hospital of Qingdao University, Taian, China

**Keywords:** CRRT, injection, pharmacokinetic, sulphobutylether beta-cyclodextrin, therapeutic drug monitoring, voriconazole

## Introduction

Voriconazole is a widely used anti-fungal drug with narrow therapeutic window (McCreary et al., 2023). It has shown large inter-individual variation in pharmacokinetics. Given the special pathophysiological condition of intensive care patients, the variation in voriconazole exposure would be even larger (Hinze et al., 2024). Thus, injection formulation could avoid absorption variation and is preferred in ICU. Continuous renal replacement therapy (CRRT) is a common organ support treatment for ICU patients, and it is challenge for dosing voriconazole for these patients. Recently, Wang et al. developed a population pharmacokinetic model of voriconazole injections for patients with pulmonary aspergillosis, and CRRT was a covariate of the final model (Wang et al., 2025). Another earlier study also included CRRT as covariate for voriconazole PPK model (Wang et al., 2024). These evidences would guide voriconazole dosing in patients receiving CRRT. However, we would like to share our opinion about voriconazole injection use in these patients.

## Formulation and pharmacokinetic considerations of voriconazole injection application in CRRT patients

Owing to the low water solubility of voriconazole, sulphobutylether beta-cyclodextrin (SBECD) was added as a solubilization agent in its injection formulation. Each bottle of voriconazole injection contained approximately 3200 mg of SBECD. As SBECD could accumulate in patients with renal impairment, patients with creatinine clearance <50 mL/min were advised to receive an oral formulation. According to its insert, the clearance rates of voriconazole and SBECD during hemodialysis were 121 mL/min and 55 mL/min, respectively. These findings indicate that 4-h hemodialysis can only remove small parts of voriconazole and SBECD. Real-world evidence concerning SBECD clearance by CRRT is limited. A previous study in three intensive patients receiving intravenous voriconazole and intermittent dialysis therapy revealed that the SBECD plasma concentrations were greater than 400 mg/mL (von Mach et al., 2006). A subsequent case series also detected SBECD accumulation in four critically ill patients with acute kidney injury under extended daily dialysis (Burkhardt et al., 2010). The trough concentrations of SBECD on day 5 were several times higher than those on day 1. Hafner et al. performed a three-period randomized crossover PK study of 15 patients with end-stage renal failure during treatment with two hemodialysis systems and hemodiafiltration, and found that SBECD recoveries in dialysate samples were 67% of the administered doses (Hafner et al., 2010). Although the half-life during renal replacement therapy nearly normalized and SBECD could be effectively eliminated by 6 h of renal replacement therapy via all methods, the prediction results indicated that SBECD still exceeded the exposure of patients with normal renal function by a factor of 6.2 in the steady state. Kiser et al. also performed an *in vivo* study in 10 patients receiving CVVH, and reported that CVVH accounted for 86% of the total body clearance of SBECD, with the majority of the dose being recovered in the effluent (Kiser et al., 2015). They reported that standard dosages of intravenous voriconazole can be utilized in patients undergoing CVVH without a significant risk of SBECD accumulation. However, some experts have suggested that the results should be interpreted with caution in different scenarios (Honore et al., 2015). Thus, caution should always be taken when intravenous voriconazole is dosed to CRRT patients to avoid SBECD accumulation.

## Clinical evidence of the safety of voriconazole injection in patients receiving CRRT

Considering the difficulty in assessing renal function fluctuations in CRRT patients, there are few data concerning the safety of intravenous voriconazole. However, there is some evidence in patients with renal impairment. Lashof et al. performed a retrospective study to evaluate the safety and tolerability of intravenous voriconazole in 41 patients with baseline renal insufficiency (Oude Lashof et al., 2012). The median duration of intravenous voriconazole treatment was 7 days. Worsening of renal function or newly emerged renal adverse events were reported in 39% of voriconazole-treated patients but were lower than those associated with the alternative drug amphotericin B. In contrast, Lilly et al. evaluated the safety of IV voriconazole compared with two other IV antifungals not containing SBECD in patients with compromised renal function (baseline Clcr <50 mL/min), and formulation with SBECD was not a predictor of AKI (Lilly et al., 2013). They concluded that the decision on which antifungal to use should not be determined by the incorporation of SBECD in the IV formulation. However, the sample size was also limited (19 patients received voriconazole). Kim also performed a prospective observation study in 25 patients (7 patients with baseline Clcr <50 mL/min) and did not find a high ADR incidence of SBECD-containing formulations (Kim et al., 2016).

The argument about intravenous voriconazole safety in patients with renal impairment, as well as in patients receiving CRRT, continues until convincing evidence emerges (Tragiannidis and Groll, 2022; Wang and Zhang, 2022).

## Comparison of voriconazole PPK parameters in CRRT patients

We searched the literature concerning the PK parameters of voriconazole under CRRT, excluding case reports. The data are shown in Table 1. The design of these studies has several limitations, such as a limited sample size or limited sampling points. The PK parameter estimates varied greatly among studies. Multiple factors, such as CRRT modality and patient characteristics, could influence voriconazole clearance and contribute to the variance in voriconazole concentrations. Unfortunately, most studies have not clarified the modality and parameter of CRRT. From the limited data we could see that three studies reported comparable clearance of voriconazole (Grensemann et al., 2021; Wang et al., 2024; Wang et al., 2025), but a study focused on continuous veno-venous hemodiafiltration (CVVHDF) modality reported a much high clearance and may have additional drug removal (Fuhrmann et al., 2007). Current models may be insufficient to estimate voriconazole exposure precisely in CRRT patients, and therapeutic monitoring may be the only way to ensure efficacy and avoid toxicity.

**TABLE 1:** Pharmacokinetic parameter estimates of voriconazole under CRRT.

Study	Patient population	Voriconazole formulation	Sampling strategy	Final model information	PK parameter estimates	CL under CRRT
Wang et al. (2025)	72 patients (150 concentration levels) with pulmonary aspergillosis; 25 of the patients were with CRRT.	All injection	Sparse sampling	1-CMT; CRRT, C-reactive protein, gamma-glutamyl transpeptidase, aspartate aminotransferase, and platelet count were covariates on CL	CL: 3.17 L/h V: 135 L	CL: 5.13 L/h
Wang et al. (2024)	408 critically ill patients with 746 voriconazole levels. CRRT was administered to 104 patients at 185 concentrations	Injection and oral formulation. 278 patients received injection. It is unspecified whether CRRT patients received voriconazole injection	Sparse sampling	2-CMT; CRRT, qCRP, CLCR, PLT, and PT were covariates on CL	CL: 3.55 L/h Vc: 33.50 L Vp: 138 L Q: 52.80 L/h	CL: 5.18 L/h
Grensemann et al. (2021)	Fifteen patients with or without liver failure. All patients receiving RRT (CVVHD or CVVH)	Injection, hydroxypropyl-beta-cyclodextrin as solubilizer	Intensive sampling	2-CMT; no covariates included for final model	CL_BODY_ = 4.70 L/h V1: 80.6 L V2: 106 L Q: 62.1 L/h	CL_RRT_ = 1.46 L/h^a^
Fuhrmann et al. (2007)	9 critic ill patient undergoing CVVHDF	Injection	Intensive sampling	2-CMT; classic PK analysis with WinNonlin	CL: 12.9 L/h V: 228 L	CL_CVVHDF_: 1.1 L/h^a^

^a^ CL, was calculated separately.

Abbreviations: CRRT, continuous renal replacement therapy; CVVH, continuous veno-venous hemodiafiltration; CVVHD, continuous veno-venous hemodialysis; CVVHDF, continuous veno-venous hemodiafiltration; PK, pharmacokinetic; CMT, compartment; CL, clearance; V, distribution volume; Q, intercompartment clearance; CLCR, creatinine clearance; PLT, platelet count; PT, prothrombin time.

## Practical considerations for clinicians

Voriconazole injection could be used in patients receiving CRRT after risk-benefit evaluation. Due to the large inter-individual variability of voriconazole and additional removal by CRRT (especially CVVHDF), therapeutic drug monitoring (TDM) should be performed to ensure optimal exposure of voriconazole. Moreover, there is a risk of SBECD accumulation for these patients and TDM for SBECD is not feasible for most institutes. Thus, symptoms and biomarkers related to kidney injury should be closely monitored, and the duration of voriconazole injection treatment should not be prolonged. Otherwise, alternate antifungals should be considered.

In conclusion, evidence concerning voriconazole injection in patients receiving CRRT is insufficient. Well-designed trials addressing this issue are needed. Caution should be taken when dosing voriconazole injections in these patients.
